# Characterization of the Microenvironment of Nodular Lymphocyte Predominant Hodgkin Lymphoma

**DOI:** 10.3390/ijms17122127

**Published:** 2016-12-16

**Authors:** Lydia Visser, Rui Wu, Bea Rutgers, Arjan Diepstra, Anke van den Berg

**Affiliations:** Department of Pathology and Medical Biology, University of Groningen, University Medical Center Groningen, 9700RB Groningen, The Netherlands; ruiwu@mail.med.upenn.edu (R.W.); b.rutgers@umcg.nl (B.R.); a.diepstra@umcg.nl (A.D.); a.van.den.berg01@umcg.nl (A.v.d.B.)

**Keywords:** microenvironment, nodular lymphocyte predominant Hodgkin lymphoma, T-cells, flow cytometry

## Abstract

Nodular lymphocyte predominant Hodgkin lymphoma (NLPHL) is characterized by a low percentage of neoplastic lymphocyte predominant (LP) cells in a background of lymphocytes. The goal of this study is to characterize the microenvironment in NLPHL. Ten NLPHL cases and seven reactive lymph nodes (RLN) were analyzed by flow cytometry for the main immune cells and multiple specific subpopulations. To discriminate between cells in or outside the tumor cell area, we used CD26. We observed significantly lower levels of CD20+ B-cells and CD56+ NK cells and higher levels of CD4+ T-cells in NLPHL in comparison to RLN. In the subpopulations, we observed increased numbers of PD-1+CD4+ T follicular helper cells (TFH), CD69+CD4+ and CD69+CD8+ T-cells and CCR7-CD45RA-CD4+ effector memory T-cells, while FoxP3+CD4+ T regulatory cells (Tregs) and CCR7-CD45RA+ terminally differentiated CD4+ T-cells were decreased in NLPHL compared to RLN. CD69+ cells were increased in the tumor cell area in CD4+ and CD8+ T-cells, while FoxP3+CD25+CD4+ Tregs and CD25+CD8+ T-cells were significantly increased outside the tumor area. Thus, we show a markedly altered microenvironment in NLPHL, with lower numbers of NK cells and Tregs. PD-1+CD4+ and CD69+ T-cells were located inside, and Tregs and CD25+CD8+ cells outside the tumor cell area.

## 1. Introduction

Hodgkin Lymphoma (HL) is a unique type of B-cell lymphoma characterized by presence of a minority of neoplastic cells (less than 1%) in a background of infiltrating reactive cells [1]. The microenvironment is considered to be shaped by the neoplastic cells and provides survival signals for the neoplastic cells and protection against anti-tumor immune responses [2].

Based on differences in histopathology and neoplastic cells, HL is classified in two subgroups: classical (c)HL and nodular lymphocyte predominant Hodgkin lymphoma (NLPHL). CHL accounts for 95% of all HL cases, whereas NLPHL accounts for only 5% of all cases [1]. Both the neoplastic cells of NLPHL, i.e., the lymphocyte predominant (LP) cells, and the composition of the cells present in the microenvironment of NLPHL are different from cHL. Increased numbers of CD4+CD57+ [3], CD4+PD-1+ [4], CD4+CD57+PD-1+ [5], and CD4+CD8+ [6] T-cells have been specifically reported in the microenvironment of NLPHL. In cHL increased numbers of Tregulatory (Treg) cells [7,8], and an increased number of T helper (Th)2 [3] and Th1 [9] cells has been reported.

The infiltrating cells located in the close vicinity of the LP cells might be the most important cells for providing survival signals and for protection against anti-tumor responses. These cells have lost expression of CD26 [10], and this characteristic can be used to distinguish them from cells that are not in the close vicinity of the LP cells in a flow cytometric analysis. A comprehensive characterization of the microenvironment, including Th cell subpopulations such as Th1, Th2, Treg and T follicular helper (TFH) cells might help to elucidate the putative interactions between the microenvironment and LP cells that play a role in the pathogenesis of NLPHL.

In this study, we analyzed 47 immune cell subpopulations to determine differences between NLPHL and reactive lymph node (RLN) by flow cytometry. Within NLPHL, changes in cell populations were also determined between CD26- and CD26+ cells, as a marker for cells within and outside the tumor cell area. Immunohistochemistry staining was performed to verify the flow cytometry results.

## 2. Results

### 2.1. Comparison of Total Cell Populations between NLPHL and RLN

**Figure 1:**
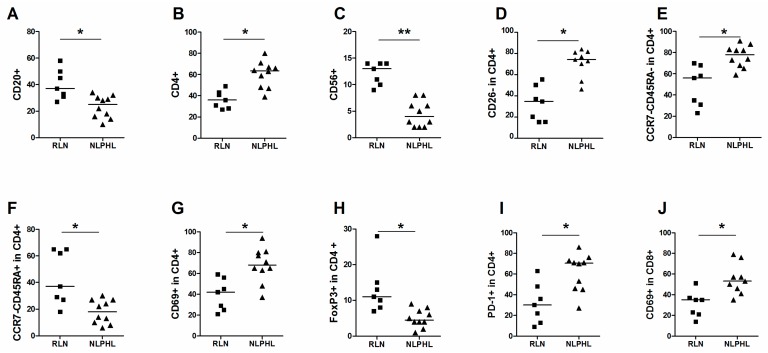
Comparison of cell populations in RLN and NLPHL. Median percentage of each group and significant changes according to p-value (0.001 < * <0.01, ** < 0.001) are indicated in each graph. Main cell populations of (**A**) CD20+; (**B**) CD4+ and (**C**) CD56+ cells. Subpopulations (**D**) CD26- in CD4+ cells; (**E**) CCR7-CD45RA- in CD4+ cells; (**F**) CCR7-CD45RA+ in CD4+ cells; (**G**) CD69+ in CD4+ cells; (**H**) FoxP3+ in CD4+ cells; (**I**) PD-1+ in CD4+ cells; and (**J**) CD69+ in CD8+ cells.

In the main cell populations, a significantly lower level of CD20+ cells was observed in NLPHL (median 25%) in comparison to RLN (median 37%) (Figure 1A). The percentage of CD4+ cells was significantly higher in NLPHL than in RLN (median 62% and 36%) (Figure 1B). The number of CD56+ NK-cells was significantly lower in NLPHL compared to RLN (4%–13%) (Figure 1C). The percentages of CD3+, CD8+ and CD68+ cells were not significantly different between RLN and NLPHL (Table 1).

### 2.2. Comparison of Subpopulations between NLPHL and RLN

The percentage of CD26- cells in the CD4+ population is significantly higher in NLPHL (median 35% vs. 74%) (Figure 1D). Within CD4+ cells, the percentage of CCR7-CD45RA-T effector memory (TEM) was significantly increased in NLPHL compared to RLN (median 56% vs. 78%) (Figure 1E), while the percentage of CD45RA+CCR7- terminally differentiated T-cells (TEMRA) was significantly lower in NLPHL compared to RLN (median 37% vs. 18%) (Figure 1F). Moreover, significantly increased percentages of CD69+ cells (median 68% vs. 42%) (Figure 1G), decreased Foxp3+ cells (median 11% vs. 4.5%) (Figure 1H) and increased PD-1+ (also known as CD279) cells (median 71% vs. 30%) (Figure 1I) were observed in CD4+ cells of NLPHL compared to RLN. In CD8+ cells, the only significant difference was observed in CD69+ cells, with a higher percentage in NLPHL compared to RLN (median 53% vs. 35%) (Figure 1J). The other subpopulations did not show significant differences between NLPHL and RLN (Table 1).

### 2.3. Comparison of Subpopulations of Cells within CD26- and CD26+ of NLPHL

**Figure 2:**
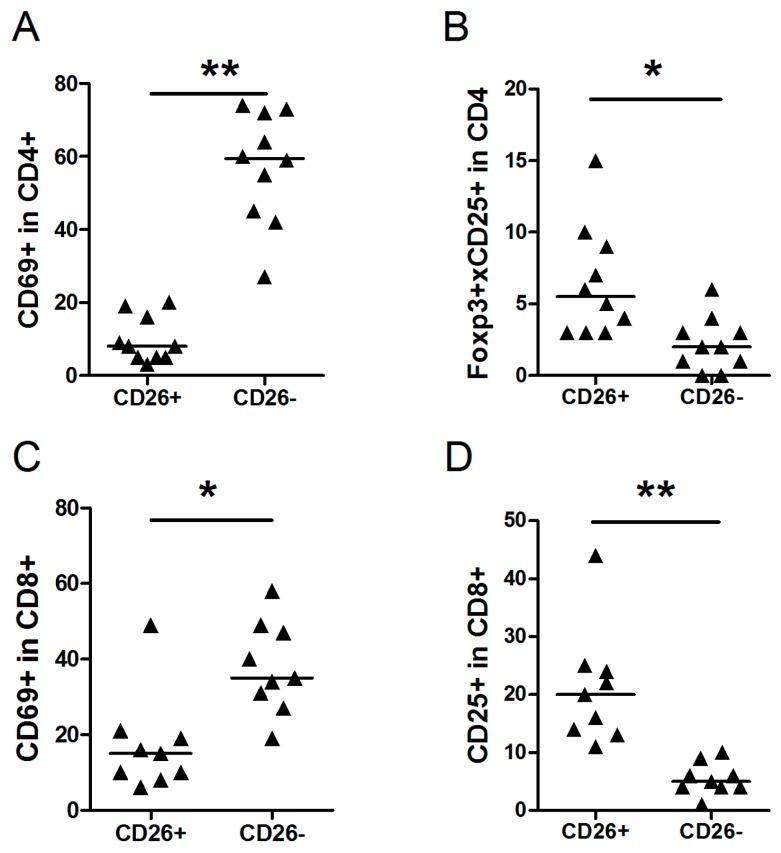
Comparison of cell populations in CD26+ and CD26- cells in NLPHL cases. Median percentage of each group and significant changes according to *p*-value (0.001 < * < 0.01, ** <0.001) are indicated in each graph. (**A**) CD69+ in CD4+ cells; (**B**) Foxp3+CD25+ in CD4+ cells CD4+; (**C**) CD69+ in CD8+ cells; and (**D**) CD25+ in CD8+ cells.

To discriminate between T-cells inside (CD26-) and outside (CD26+) the tumor cell area in NLPHL, we co-stained T-cell subsets with CD26. The percentage of CD4+CD69+ cells in CD26- cells (median 60%) was significantly higher compared to the percentage of CD4+CD69+ cells in CD26+ cells (median 8%) (Figure 2A). A significantly lower percentage of Foxp3+CD25+CD4+ cells was observed in CD26- cells compared to CD26+ cells (median 2% vs. 5%) (Figure 2B). Significantly higher percentages of CD8+CD69+ cells were found in CD26- compared to CD26+ cells (median 34% vs. 15%) (Figure 2C). Significantly lower percentages of CD8+CD25+ were detected in CD26- cells compared to CD26+ cells (median 5% vs. 20%) (Figure 2D). The other subpopulations did not show any significant differences (Table 2).

### 2.4. Immunohistochemical Staining of NLPHL

**Figure 3:**
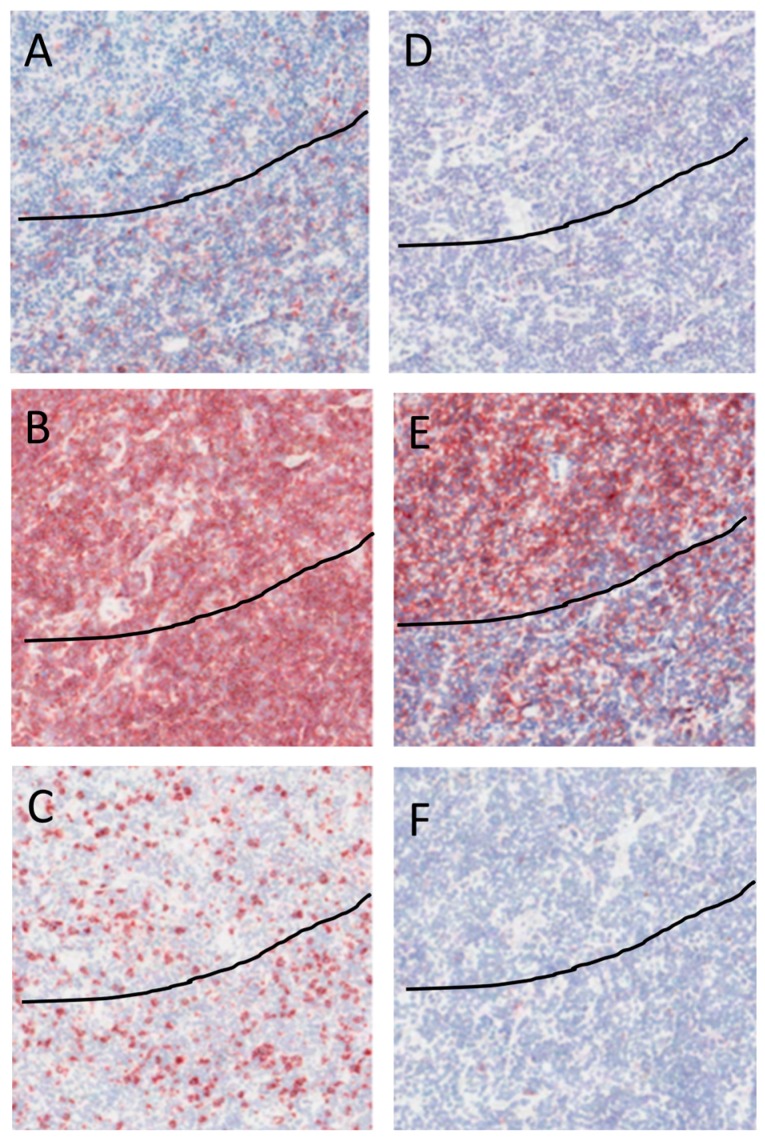
Immunohistochemistry of a representative NLPHL case. All figures show the same area of tumor with magnification 40×. The line discriminates between the tumor cell rich area, i.e., area above line, from the tumor cell depleted area. Positive staining is visualized in red, counterstaining of the nucleus with hematoxylin is blue. (**A**) CD26+ cells are found mainly outside the tumor area; (**B**) CD4+ cells are scattered all over the tissue; (**C**) CD8+ cells are scattered all over the tissue; (**D**) No expression of Foxp3 in the cells in tumor area, few Foxp3 positive cells are present outside of the tumor area; (**E**) Aggregation of CD69 stained cells in the tumor area; and (**F**) Few CD25 stained cells in the tumor area and out of the tumor area.

Immunohistochemical staining with CD26 revealed rare CD26+ cells in the nodules that contain the tumor cells, while the number of CD26+ cells was high outside the tumor cell areas (Figure 3A). CD4+ cells were scattered in high numbers all over the tissue both in and outside the tumor cell area (Figure 3B). CD8 cells showed a similar distribution pattern as CD4+ cells albeit at lower numbers (Figure 3C). A low number of Foxp3+ cells was observed out of the tumor cell area, while no FoxP3+ cells were detected in the tumor cell area (Figure 3D). CD69+ cells were present at higher numbers in the tumor cell areas as compared to outside the tumor cell areas (Figure 3E). A few CD25+ cells were present both in and out the tumor cell areas (Figure 3F). The immunohistochemistry results were consistent with the findings by flow cytometry using CD26 to discriminate between cells within and outside the tumor cell areas.

## 3. Discussion

The composition of the cell types in the microenvironment of NLPHL and their role in the survival of LP cells are less studied than the composition and relevance of the cells in the microenvironment of cHL. The goal of this study was to generate a comprehensive overview of the cell types present in the microenvironment of NLPHL. We detected a decrease in the number of B-cells and an increase in CD4+ T-cells compared to RLN, which has been reported previously [11]. The number of NK cells was decreased in NLPHL. NK cells along with macrophages form the innate immune response against tumor cells. A reduced number of NK cells might thus contribute to the failure of the immune system to eradicate the LP cells. The lower number of NK cells in NLPHL compared to RLN has not been reported previously. Consistent with a previous study showing an increased percentage of double positive CD4+CD8+ T-cells in NLPHL [6], we also observed an increase in our data (3% in RLN to 8% in NLPHL), although this did not reach significance.

The subpopulation that showed the most prominent increase in NLPHL compared to RLN is PD-1+CD4+ T-cell subset, and these cells have been reported to surround the LP cells [4]. We have previously described the phenotype of these TFH cells, which are PD-1+, partially CD57+ and BCL-6 positive [5]. Since the IL21 receptor is upregulated in LP cells [12], and IL-21 is produced by TFH cells, these cells might be beneficial for survival of LP cells. The decreased number of FoxP3+ Tregs in the tumor cell area might explain the increased numbers of TFH cells, as Tregs control the number of TFH cells [13]. Of the different marker combinations we used to analyze the percentages of TFH cells, we observed a significant increase only in PD-1+CD4+ cells (30 and 70%) and no change in the percentages of CXCR5+ICOS+CD4+ (8% and 5%) and CXCR5+BCL6+CD4+ (12% and 13%) cells in NLPHL. LP rosetting cells have been reported to be negative for the inducible T-cell co-stimulator (ICOS) by IHC [14]. Expression of ICOS in TFH cells is important for maintaining expression of CXCR5 and BCL6 and for maintaining their location in the germinal center [15,16]. Thus, our data suggest that lack of ICOS expression in the LP rosetting PD-1+CD4+ cells NLPHL might explain their unusual phenotype. How these cells lose ICOS expression and whether LP cells play a role in this phenomenon is not known. On the other hand, PD-1 also is a marker of T-cell exhaustion, and it is possible the PD-1+CD4+ cells in NLPHL are not TFH cells but exhausted CD4+ cells. Treatment with anti-PD-1 has shown promising effects in several tumors, including cHL [17] and might possibly also be effective in NLPHL, although PD-L1 is not expressed in LP cells [18]. The level of PD-1 expression has been shown to distinguish between TFH cells (high expression) and exhausted T-cells (low expression) [19]. The PD-1+CD4+ cells in NLPHL show high PD-1 expression [4,5] and are mostly likely not exhausted. So, the nature of these PD-1+CD4+ cells in NLPHL remains unclear.

We found an increase in the population of CCR7-CD45RA-CD4+ TEM cells in NLPHL compared to RLN. TEM cells are present in peripheral blood and, upon infection, home to peripheral tissues to initiate an inflammatory response [20,21]. The high level of both TEM and PD-1+CD4+ cells in NLPHL suggests that at least a part of the TEM cells overlap with PD-1+CD4+ cells. Our flow cytometry and IHC results indicate a significant increase in CD69+CD4+ cells in the tumor cell areas. In addition to being an early activation marker, CD69 expression also inhibits the egress of CD4+ T-cells from lymphoid organs, and is expressed on tissue remaining memory cells (TRM) [22]. The increased percentages of TEM and PD-1+CD4+ cells in NLPHL, combined with the increased numbers of CD69+ cells in the tumor cell area, suggests that egress of these cells from the tumor cell area is inhibited [23]. Thus, these putative CD69/PD-1 double positive cells might in fact represent TRM cells. CD69+ cells have been proposed to downregulate autoimmunity by producing TGF-β [24]. Vice versa, loss of CD69+ cells and thus loss of TGF-β production can result in enhanced anti-tumor responses [25]. These data suggest that the presence of CD69+ T-cells in the tumor cell area of NLPHL has an immune suppressive effect by producing TGF-β. TGF-β was indeed present in NLPHL derived CD4+CD57+, CD4+CD57- and CD4-CD57- cells at the mRNA level, but the levels were not different from tonsil derived T-cells, in which the number of CD69+ cells are lower [3]. As fibrosis normally caused by TGF-β is not seen in NLPHL, further studies to elucidate the actual role of these cells in NLPHL are required.

The number of Foxp3+CD25+CD4+ cells is significantly lower within the tumor cell area as compared to the number outside the tumor cell area. This is different from cHL where an increased number of Tregs was found within the tumor cell area [26]. This suggests different anti-tumor escape mechanisms in both HL subtypes.

CD69+CD8+ cells are present in the tumor cell area, whereas outside the tumor area CD8+ cells expressed the late activation marker CD25+. Presence of early-activated CD8+ cells that lack expression of CD25 in the tumor cell areas of NLPHL might explain why CD8+ cells are not able to eradicate the neoplastic cells. The CD25+CD8+ cells are found especially outside the tumor cell area where Tregs are present in somewhat higher numbers to downregulate putative immune responses.

In conclusion, Tregs and NK cells are decreased in NLPHL compared to RLN. LP cells in NLPHL are surrounded by PD-1+CD4+ and CD69+CD4+ cells with a TEM phenotype, while levels of Tregs and CD25+CD8+ cells are increased outside the tumor cell area.

## 4. Methods and Materials

### 4.1. Patients

Cell suspensions of RLN (*n* = 7) and NLPHL (*n* = 10) were obtained from fresh tissue and stored in liquid nitrogen. The age of the patients was not significantly different between RLN (mean 43, range 17–72) and NLPHL (mean 36, range 6–75), neither was the gender (RLN 4 males (56%) and NLPHL 7 males (70%)). The study protocol was consistent with international ethical and professional guidelines (the Declaration of Helsinki and the International Conference on Harmonization Guidelines for Good Clinical Practice). The use of anonymous remnantmaterial is regulated under the code for good clinical practice in the Netherlands. Informed consent was waived in accordance with Dutch regulations.

### 4.2. Flow Cytometry

For flow cytometry, cell suspension of 7 samples from RLN and 10 samples of NLPHL were used. 0.5 × 10^6^ cells were incubated with different mixes of fluorescent labeled antibodies (Table S1) for 30 min in the dark at 4 °C. For intracellular staining, cells were treated with fixation/permeabilization buffer (E-biosciences, San Diego, CA, USA) for 30 min, followed by incubation with permeabilization buffer containing 5% human serum for 15 min, before incubation with the primary antibodies. Fixation of the cells was done with 2% paraformaldehyde in PBS. Unstained samples were used to set gating for membrane markers and isotype controls were used for intracellular labeling. All samples were analyzed on the BD FACSCalibur (BD, Franklin Lakes, NJ, USA) and the Winlist software package (Verity Software House, Topsham, ME, USA) was used for data analysis.

### 4.3. Immunohistochemistry

Frozen tissue sections of 4 RLN and lymph nodes of 10 NLPHL patients were used for immunostaining of selected markers. After fixation with acetone, CD4 (1:10), CD8 (1:10), CD25 (1:20) (IQ Products, Groningen, The Netherlands), CD69 (1:100), Foxp3 (1:100) (Abcam, Cambridge, UK) and CD26 (undiluted, our lab) antibodies were incubated for 60 min. Secondary (polyclonal rabbit anti mouse immunoglobulin horseradish peroxidase labeled, 1:100) and tertiary (polyclonal goat anti rabbit immunoglobulin horseradish peroxidase labeled, 1:100) (Dako, Glostrup, Denmark) antibody incubation steps in PBS with 1% human serum were performed for one hour. Visualization was done using 3-Amino-9-ethylcarbazole as a substrate for peroxidase. Slides were counterstained with Mayer’s hematoxylin. Slides were scored for the positive staining in and outside the tumor area.

### 4.4. Statistics

The SPSS software package (version 22, IBM, Amsterdam, The Netherlands) was used for statistical analysis. Differences in age and gender between RLN and NLPHL patient groups were determined by Mann-Whitney test and Fisher exact test respectively. Flow cytometry results were analyzed by a Mann-Whitney test to assess significant differences between the two groups. To correct for multiple testing of subpopulations, which were at least in part dependent, we considered *p* < 0.01 as being statistically significant.

## Figures and Tables

**Table 1:** Median percentages of cell populations analyzed in NLPHL and RLN.

Cell Type	Population	Median % (Range)				*p*-Value
		RLN		NLPHL		
Main cell populations	CD20+ in live	37	27–58	25	10–34	0.007
	CD3+ in live	57	39–65	76	46–89	0.017
	CD4+ in live	36	29–51	62	41–71	0.002
	CD8+ in live	22	15–40	20	13–27	0.1
	CD56+ in live	13	9–14	4	2–8	0.00005
	CD68+ in live	2	1–2	1.5	1–3	1
	CD4+CD8+ in live	3	2–9	8	3–17	0.016
CD26-	CD26- in CD4+	35	15–56	74	46–84	0.0012
	CD26- in CD8+	34	26–61	34	26–72	0.2439
Maturation of CD4+	Naive (CCR7+CD45RA+ in CD4+)	6	3–11	2.5	1–7	0.016
	TCM (CCR7+CD45RA- in CD4+)	1	0–2	2	1–8	0.08
	TEM (CCR7-CD45RA- in CD4+)	56	23–70	78	59–91	0.002
	TEMRA (CCR7-CD45RA+ in CD4+)	37	18–65	18	6–30	0.007
Activation of CD4+	CD69+ in CD4+	42	21–59	68	37–94	0.003
	CD25+ in CD4+	10	5–21	11	4–18	0.5
Th1	CXCR3+ in CD4+	18	11–56	15	8–33	0.3
Th2	ST2L+ in CD4+	14	4–23	9	4–18	0.3
	CXCR4+ in CD4+	5	3–14	6	3–9	1
Treg	GITR+ in CD4+	17	10–66	33	15–43	0.3
	GITR+CD25+ in CD4+	6	2–11	8.5	5–17	0.2
	CD127- in CD4+	15	5–31	24	19–56	0.02
	CD127-CD25+ in CD4+	2	1–3	1	1–4	0.8
	CD152+ in CD4+	8	5–17	6.5	3–9	0.3
	CD152+CD25+ in CD4+	7	3–13	4.5	2–6	0.4
	FoxP3+ in CD4+	11	7–28	4.5	1–9	0.001
	CD25+FoxP3+ in CD4+	4	2–11	2.5	1–5	0.1
	CD25+CD45RA- inCD4+	4	0–6	4	0–11	0.1
TFH	CD57+ in CD4+	8	3–22	21	3–39	0.1
	PD-1+ in CD4+	30	9–63	71	27–86	0.009
	PD-1+CD57+ in CD4+	7	2–21	20	2–38	0.2
	CXCR5+ICOS+ in CD4+	8	5–27	5	1–15	0.017
	CXCR5+ X ICOS+ in CD25+/CD4+	31	18–50	26	11–36	0.4
	Bcl6+ in CD4+	15	3–35	25	4–40	0.2
	CXCR5+BCL6+ in CD4+	12	2–30	13	1–30	0.7
	Bcl6+CD57+ in CD4+	7	3–15	11	3–15	0.1
Cytotoxic CD4+	TIA-1+ in CD4+	7	1–13	15	3–32	0.012
	Granzyme-B+ in CD4+	2	1–5	3	1–4	0.8
Activation of CD8+	CD25+ in CD8+	30	12–32	27	14–51	0.6
	CD69+ in CD8+	35	14–51	53	35–79	0.005
CD8+	CXCR4+ in CD8+	30	21–44	33.5	10–78	0.8
	CXCR3+ in CD8+	41	20–63	33	21–44	0.2
	TIA-1+ in CD8+	25	19–49	40.5	28–72	0.03
	Granzyme-B+ in CD8+	6	4–27	11	4–21	0.04
NK/NKT	CD56+ in CD3-	17	15–26	4	1–22	0.8
	CD16+ in CD3-	13	6–24	8.5	3–21	0.2
	CD57+ in CD3-	4	1–8	5	3–16	0.1
	CD56+CD16+ in CD3-	4	1–11	1.5	1–6	0.1
	CD56+CD107a+ in CD3-	1	1–1	1	0–2	0.2
	CD56+CD16+ in CD3+	4	2–10	2	1–5	0.04
Macrophage	CD163+ in CD68+	17	7–32	19	12–36	0.1

Th1: T helper 1; Th2: T helper 2; Treg: T regulatory; TFH: T follicular helper; NK/NKT: natural killer/natural killer T; TCM: central memory T; TEM: effector memory T; TEMRA: terminally differentiated.

**Table 2:** Comparison of median percentages of CD26+ and CD26- cell populations in NLPHL.

Cell Type	Median % [Range]				*p*-Value
	CD26+		CD26-		
CD69+ in CD4+	8	3–20	60	27–74	0.00001
CD25+ in CD4+	5	2–11	3	1–7	0.2
FoxP3+ in CD4+	2	1–3	4	0–7	0.1
Foxp3+CD25+ in CD4+	5	3–15	2	0–6	0.003
CXCR3+ in CD4+	4	2–12	7	3–21	0.017
ST2L+ in CD4+	3.5	2–6	4.4	2–12	0.3
CXCR3+ in CD8+	17	10–29	19	10–29	0.5
CD69+ in CD8+	15	6–49]	34	19–58	0.004
CD25+ in CD8+	20	11–44	5	1–10	0.00004

## Supplementary Materials

Supplementary materials can be found at www.mdpi.com/1422-0067/17/12/2127/s1.
