# Erratum: Lydia Visser et al. Characterization of the Microenvironment of Nodular Lymphocyte Predominant Hodgkin Lymphoma, *Int. J. Mol. Sci.* 2016, 17, 2127

**DOI:** 10.3390/ijms19010304

**Published:** 2018-01-19

**Authors:** Ahmad Sattarzadeh, Lydia Visser, Bea Rutgers, Arjan Diepstra, Anke van den Berg

**Affiliations:** Department of Pathology and Medical Biology, University of Groningen, University Medical Center Groningen, 9700RB Groningen, The Netherlands; a.sattarzadeh@umcg.nl (A.S.); b.rutgers@umcg.nl (B.R.); a.diepstra@umcg.nl (A.D.); a.van.den.berg01@umcg.nl (A.v.d.B.)

The authors regret to have made a mistake in publishing this paper [1] with an incorrect author list. The problem was caused by miscommunication between the listed authors and Ahmad Sattarzadeh. When we got in touch with him to discuss this matter, the paper was already published. As Ahmad Sattarzadeh performed all analyses and wrote the paper as part of his thesis, we added Ahmad Sattarzadeh as the first author after a careful discussion with all the authors; Rui Wu was removed from the list since her contribution—setting up the analysis for the flowcytometry—did not justify an authorship on this paper. So, the author list has changed from “Lydia Visser *, Rui Wu, Bea Rutgers, Arjan Diepstra, Anke van den Berg” to “Ahmad Sattarzadeh, Lydia Visser *, Bea Rutgers, Arjan Diepstra, Anke van den Berg”.

The change does not affect the scientific results. The manuscript will be updated and the original will remain online on the article webpage, with a reference to this Erratum.
