# Supplementary material for: Characterization of the Microenvironment of Nodular Lymphocyte Predominant Hodgkin Lymphoma
Source: Int J Mol Sci. 2016 Dec 16;17(12):2127. doi: 10.3390/ijms17122127 (PMC5187927; doi:10.3390/ijms17122127)
Supplement: Supplementary file 1 [file ijms-17-02127-s001.pdf]

Supplementary Materials: Characterization of the Microenvironment of Nodular Lymphocyte Predominant Hodgkin Lymphoma

Lydia Visser, Rui Wu, Bea Rutgers, Arjan Diepstra and Anke van den Berg

Table S1. Leukocyte subpopulations and antibodies used.

Leukocyte Subpopulation	Immunophenotype	Company
B-cells	CD20+	BD
T-cells	CD3+	Dako
Th cells	CD4+	BD
Cytotoxic T-cells	CD8+	BD
NK cells	CD56+	IQ
Macrophages	CD68+	R&D
Double positive T-cells	CD4+CD8+	BD/BD
Naïve Th cells	CCR7+CD45RA+ in CD4+	R&D/Own lab/BD
Central memory Th cells (TCM)	CCR7+CD45RA− in CD4+	R&D/Own lab/BD
Effector memory Th cells (TEM)	CCR7-CD45RA− in CD4+	R&D/Own lab/BD
Terminally differentiated Th cells (TEMRA)	CCR7-CD45RA+ in CD4+	R&D/Own lab/BD
Activation of Th cells	CD69+ in CD4+	IQ/BD
	CD25 in CD4	IQ/IQ
Th1 cells	CXCR3+ in CD4+	R&D/IQ
Th2 cells	ST2L+ in CD4+	R&D/BD
	CXCR4+ in CD4+	R&D/IQ
Regulatory T-cells	GITR+ in CD4+	R&D/BD
	GITR+ CD25+ in CD4+	R&D/I Q/BD
	CD127low in CD4+	BD/BD
	CD127low CD25+ in CD4+	BD/IQ/BD
	CD152+ in CD4+	IQ/BD
	CD152+ CD25+ in CD4+	IQ/IQ/BD
	FoxP3+ in CD4 +	BD/BD
	CD25+ FOXP3 + in CD4+	IQ/BD/BD
	CD25+ CD45RA- inCD4+	IQ/Own lab/BD
T follicular helper cells	CD57+ in CD4	BD/BD
	PD-1+ in CD4	Bio/BD
	PD-1+ CD57+ in CD4	Bio/BD/BD
	CXCR5+ ICOS+ in CD4+	BD/R&D/BD
	Bcl6+ in CD4+	R&D/BD
	CXCR5+ BCL6+ in CD4+	BD/R&D/BD
	Bcl6+ CD57+ in CD4+	R&D/BD/BD
T follicular helper regulatory cells	CXCR5+ICOS+ in CD4+CD25+	R&D/BD/BD/IQ
Cytotoxic Th cells	TIA-1+ in CD4+	IQ/BC
	Granzyme-B+ in CD4+	IQ/BD
Activation of cytotoxic T-cells	CD25+ in CD8+	IQ/BD
	CD69+ in CD8	IQ/BD
Cytotoxic T-cells	CXCR4+ in CD8+	R&D/BD
	CXCR3+ in CD8+	R&D/BD
	TIA-1+ in CD8+	BC/BD
	Granzyme B+ in CD8+	BD/BD

Table S1. *Cont.*

Leukocyte Subpopulation	Immunophenotype	Company
NK cells	CD56+ in CD3−	IQ/BD
	CD16+ in CD3−	R&D/BD
	CD57+ in CD3-	BD/BD
	CD56+ CD16+ in CD3−	IQ/R&D/BD
	CD56+ CD107a+ in CD3−	IQ/BD/BD
	CD56+CD16+ in CD3+	IQ/R&D/BD
M2 Macrophages	CD163+in CD68+	R&D/R&D

BD: BD Biosciences; IQ: IQ Products, Groningen, The Netherlands; R&D: R&D systems, Minneapolis, MN, USA; Dako: Dako Products, Glostrup, Denmark; BC: Beckman Coulter, Woerden, The Netherlands.
